# Rheumatic Mitral Valve Surgery: Repair or Replacement?

**DOI:** 10.21470/1678-9741-2023-0294

**Published:** 2025-02-11

**Authors:** Luís Henrique Oliveira Pereira, Kelvin Câmara, Tamires Santos Pinheiro, Matheus Mônaco Lemos, Ana Laísa Andrada Oliveira, Maria Eduarda Pereira de Oliveira, Gabrielly Machado Trindade, Manoel Flávio Silva Kanisky, Marjorie Francisca Raksa, Gerlanio César da Silva, Valdano Manuel

**Affiliations:** 1 Department of Medicine, Faculdade de Medicina, Universidade Centro de Ensino de Maringá (UNICESUMAR), Maringá, Paraná, Brazil; 2 Faculdade Pernambucana de Saúde (FPS), Recife, Pernambuco, Brazil; 3 Department of Medicine, Faculdade de Medicina, Universidade Vila Velha (UVV), Vila Velha, Espírito Santo, Brazil; 4 Department of Medicine, Faculdade de Medicina, Centro Universitário Max Planck (UniMax), Indaiatuba, São Paulo, Brazil; 5 Clínica Girassol, Luanda, Angola; 6 Complexo Hospitalar de Doenças Cardio-Pulmonares Cardeal Dom Alexandre do Nascimento, Luanda, Angola; 7 Instituto do Coração (InCor), Hospital das Clínicas, Universidade de São Paulo, São Paulo, São Paulo, Brazil

**Keywords:** Rheumatic Heart Disease, Reoperation, Mitral Valve, Treatment Outcome, Review

## Abstract

**Introduction:**

Rheumatic heart disease remains a public health problem, especially in developing countries. The mitral valve (MV) is the main affected cardiac structure, requiring intervention in many cases. The discussion of which is the best option - repair or replacement - is still a controversy.

**Objective:**

To compare the survival of patients with rheumatic MV submitted to replacement or repair.

**Methods:**

We systematically reviewed the English literature through PubMed®, Literatura Latino-Americana e do Caribe em Ciências da Saúde (or LILACS), Scientific Electronic Library Online (or SciELO), and Google Scholar between January 2021 and February 2022, based on the Preferred Reporting Items for Systematic Reviews and Meta-Analyses (or PRISMA) methodology. Articles with a sample of at least 30 patients who underwent MV replacement or repair were included.

**Results:**

Six studies including 2874 patients were analyzed. Most of the patients were female (2001; 69.6%) with a ratio of 2.3:1. The ages ranged from 11 to 66 years. The mean follow-up varied from six to 106 months. In the MV repair group, mortality was 2.5% (62 of 2473) and reoperation was 3.7% (93 of 2473), while in the MV replacement group, mortality was 8.2% (106 of 1291), and 3.6% (54 of 1475) of the patients required reoperation. The patient's survival was similar (85% for repair and 87% for replacement). The main complications post-MV repair or replacement were stroke (1.8%; 2.5%) and endocarditis (0.5%; 1.3%).

**Conclusion:**

The MV repair had lower mortality and fewer complications compared to MV replacement. Reoperation rate and survival are similar.

## INTRODUCTION

**Table:** 

Abbreviations, Acronyms & Symbols	
ARF	= Acute rheumatic fever
IE	= Infectious endocarditis
LILACS	= Literatura Latino-Americana e do Caribe em Ciências da Saúde
MV	= Mitral valve
NYHA	= New York Heart Association
PRISMA	= Preferred Reporting Items for Systematic Reviews and Meta-analyses
QALYs	= Quality-adjusted life years
RHD	= Rheumatic heart disease
RMV	= Rheumatic mitral valve
SciELO	= Scientific Electronic Library Online

Rheumatic heart disease (RHD) is a chronic complication of rheumatic carditis, one of the main events of acute rheumatic fever (ARF). While rheumatic carditis includes a spectrum of lesions, RHD presents chronic valve lesions that evolve over the years after one or more episodes of ARF^[1]^. The main cause of death and disability from RHD is heart failure since the heart valve is progressively healed and damaged over time^[2]^.

Regarding epidemiology, high rates of RHD persist in poor regions of the world, where it remains endemic, especially in the Oceania, South Asia, and Sub-Saharan Africa. The prevalence in these regions reached more than 1,000 cases per 100,000, while in non-endemic countries it was 3.4 cases per 100,000 inhabitants^[3]^. Exposing mortality, there were 347,500 deaths from RHD in 1990 and 319,400 deaths in 2015, a decrease of 8.1%, in endemic countries. Global age-standardized mortality from RHD decreased by 47.8% from 1990 to 2015 (9.2 to 100,000 × 4.8 to 100,000). It is estimated that 77% and 82% of deaths in 1990 and 2015, respectively, occurred in places with endemic patterns of the disease^[3]^. Regarding mortality, these same regions presented high rates, being defined as a rate > 0.15 deaths per 100,000 inhabitants among children aged from five to nine years^[4]^.

It is known that RHD predominantly affects the mitral valve (MV) and induces different degrees of regurgitation, stenosis, or both^[4,5]^. In severely affected MV, surgery is the treatment of choice, that consists basically of repair or replacement with prostheses (mechanical or biological). The decision of which procedure should be chosen must consider the availability of anticoagulants, the type of bioprostheses, and whether the patient will be able to have a future reoperation^[5]^. In general, surgical mortality is around 10%, with slight variation in places with poor access to health^[5]^.

A study conducted in India compared the cost-benefits of surgical treatments for rheumatic mitral valve (RMV) disease, with the estimate of quality-adjusted life years (QALYs) suggesting that repair is the most economical surgical intervention, while the replacement by bioprosthesis had better cost-benefit^[6]^.

Many challenges are associated to the care of patients with RHD in most affected regions such as echocardiographic screening for early detection, cardiovascular care for advanced cases in low-resource settings, the effective implementation of cardiac surgery services with availability of valve procedures, and facilitating access to definitive care^[4]^.

Within this perspective, this study aims to compare the survival of patients with RMV submitted to replacement or repair, contributing to overcoming some of these challenges, since it offers a dynamic and assertive view of the surgical management of this population.

## METHODS

### Literature Search

PICOS (standing for Problem, Intervention, Comparison, and Outcomes) and FINER (standing for Feasible, Interesting, Novel, Ethical, and Relevant) criteria guided the formulation of the research question and were applied in the Preferred Reporting Items for Systematic Reviews and Meta-analyses (PRISMA) methodology^[7,8]^.

The Descriptors in Health Science used were "Rheumatic Heart Disease AND Mitral Valve Repair", "Rheumatic Heart Disease OR Mitral Repair", "Rheumatic Heart Disease AND Mitral Valve Plasty", "Rheumatic Heart Disease AND Surgery", "Rheumatic Heart Disease OR Surgery", "Rheumatic Heart Disease AND Surgical Technique", "Rheumatic Heart Disease OR Surgical Technique", "Mitral Valve Repair AND Mitral Valve Plasty", "Mitral Repair OR Surgery", "Mitral Repair AND Surgical Technique", "Mitral Repair OR Surgical Technique", "Surgery AND Surgical Technique", and "Surgery OR Surgical Technique". The databases used were PubMed®, Google Scholar, Literatura Latino-Americana e do Caribe em Ciências da Saúde (or LILACS), and Scientific Electronic Library Online (or SciELO). English Literature from January 1, 2021 to February 28, 2022 was reviewed. Seven independent researchers extracted the data. When concordance was absent, three other researchers checked the article together and made the final decision.

### Inclusion and Exclusion Criteria

For studies whose patients were under 70 years of age, all primary studies published in English that included at least 30 patients, whether observational or experimental, were included. Studies with patients older than 70 years, unpublished articles, articles that only the abstract is in English, secondary studies, papers describing the two different techniques, books or book chapters, conference papers, and clinical trial protocol were excluded^[8]^.

### Data Extraction

All data were organized in a table presented as authors, year of publication, country, type of study, sample size, mean age, sex, follow-up time, time for reoperation, mortality, mitral stenosis, New York Heart Association (NYHA), and survival^[7]^. This is a systematic review without meta-analysis.

### Data Analysis

The articles were selected based on the Newcastle Ottawa Scale (Table 1)^[9]^. From this selection, we excluded two of the eight previously selected articles, including only six studies in our review.

**Table 1:** Newcastle-Ottawa Scale.

	1) Selection				2) Comparability	3) Outcome			
Case-control study	Is the case definition adequate?	Representativeness of the cases	Selection of controls	Definition of controls	Comparability of cases and controls on the basis of the design or analysis	Ascertainment of exposure	Same method of ascertainment for cases and controls	Non-responsive rate	Total score (out of 9)
Stale Wagen Hauge et al.^[14]^	^*^	^*^	^*^	^*^	^**^	^*^			7
	1) Selection				2) Comparability	3) Outcome			
Cohort studies	Representativeness of the exposed cohort	Selection of the non-exposed cohort	Ascertainment of exposure	Demonstration that outcome of interest was not present at start of study	Comparability of cohorts on the basis of the design or analysis	Assessment of outcome	Was follow-up long enough for outcomes to occur?	Adequacy of follow-up of cohorts	Total score (out of 9)
Shibikom Tamirat et al.^[10]^	^*^	^*^	^*^	^*^	^**^	^*^	^*^	^*^	9
Emrah Usta et al.^[11]^	^*^	^*^	^*^	^*^	^**^	^*^	^*^	^*^	9
Ross L. Roberts Thomson et al.^[12]^	^*^	^*^	^*^	^*^	^*^	^*^	^*^	^*^	8
Mohamed Amr et al.^[13]^	^*^	^*^	^*^	^*^	^**^	^*^	^*^	^*^	8
Jintao Fu et al.^[15]^	^*^	^*^	^*^	^*^	^**^	^*^	^*^	^*^	9

* PRISMA 2020 flow diagram for new systematic reviews which included searches of databases and registers only. Consider, if feasible to do so, reporting the number of records identified from each database or register searched (rather than the total number across all databases/registers)^9^.

** PRISMA 2020 flow diagram for new systematic reviews which included searches of databases and registers only. If automation tools were used, indicate how many records were excluded by a human and how many were excluded by automation tools^9^.

## RESULTS

### Study Selection

**Fig. 1:**
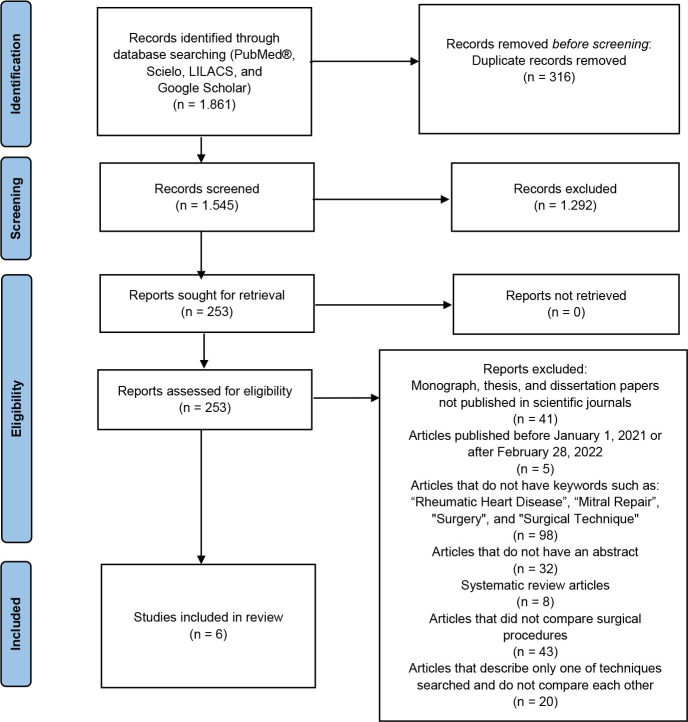
The Preferred Reporting Items for Systematic Reviews and Meta-Analyses (or PRISMA) flowchart. LILACS=Literatura Latino-Americana e do Caribe em Ciências da Saúde; SciELO=Scientific Electronic Library Online.

The PRISMA flowchart describing the search process is presented in Figure 1.

### Study Characteristics

**Fig. 2:**
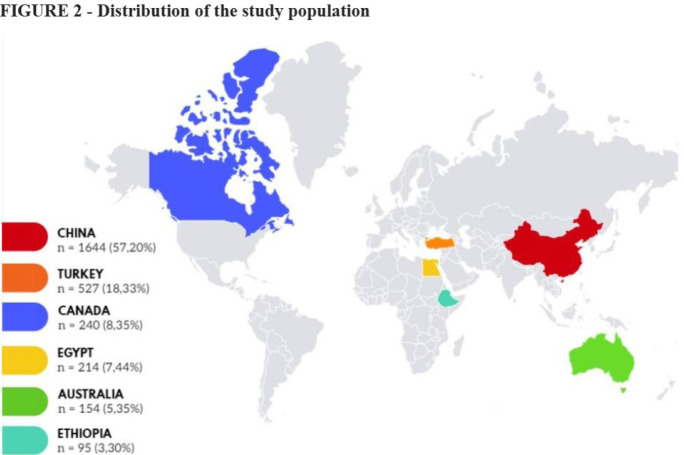
Distribution of the study population.

Six studies were included in the final analysis accounting for 2,874 patients (Figure 2). Of the six studies, four were retrospective cohorts^[10-13]^, one was a prospective cohort^[14]^, and one was a case control^[15]^. Of these, four^[10,11,13,14]^ performed a comparison between the repair and replacement methods, and the other two studies^[12,15]^ compared the first groups without and with intervention, and after compared repair and replacement, as presented in Table 2.

**Table 2:** Comparison of data between repair and replace according to selected articles.

	Mortality		Survival		Mitral stenosis		Reoperation	
	Repair	Replace	Repair	Replace	Repair	Replace	Repair	Replace
Tamirat^[10]^	4% (n=12)	8.1% (n=12)	94% (n=86)	87% (n=129)	Before: 9% (n=8)	Before: 8.7% (n=13)	2.2% (n=2)	5.4% (n=8)
Fu^[15]^	2% (n=11)	8.2% (n=73)			Before: 9.5% (n=58)			
After: 10.4% (n=55)	Before: 15% (n=155)							
After: 11.9% (n=63)	2.3% (n=13)	1.4% (n=12)						
Usta^[11]^	0% (n=0)	4% (n=10)	81% (n=206)	88% (n=222)			16.3% (n=45)	2.4% (n=6)
Roberts-Thomson^[12]^					Before: 16.5% (n=14)			
After: 14.4% (n=10)	Before: 16.5% (n=14)							
After: 14.4% (n=10)								
Hauge^[14]^	11% (n=5)		89% (n=90)				2.1% (n=2)	
Amr^[13]^	19.6% (n=42)				27% (n=58)		14% (n=30)	12% (n=26)

Most of the patients were female, with 2001 women (69.6%) and 873 men (30.4%), with a ratio of 2.3:1 for female:male. The age ranged from 10 to 66 years; in the repair group it was 10 to 65 years, and in the replacement group it was 12 to 66 years.

A total of 1058 (36.81%) patients underwent MV repair, and 1677 (58.35%) underwent replacement, thus excluding 139 (4.84%) patients who were in the group control in one study. The main repair techniques cited by the two articles were annuloplasty, commissurotomy, papillary muscle division, posterior leaflet enlargement, neocords, and subvalvular procedures. Regarding the replacement, the ratio between mechanical and bioprosthetic valves is 3:1, respectively (76.9% × 23.1%), as presented in three studies.

### New York Heart Association

Four articles described pre and postoperative NYHA class (1076 patients). Preoperatively, in the repair group, three studies reported 205 patients in NYHA classes I-II, and 305 patients in NYHA classes III-IV. The postoperative results had 377 patients in NYHA classes I-II, and 59 patients in NYHA classes III-IV. While in the replacement group, preoperative NYHA classes I-II account for 256 patients and NYHA classes III-IV for 307 patients; postoperatively, 322 patients were in NYHA classes I-II, and 24 patients in NYHA classes III-IV.

### Rate of Reoperation

The rate of reoperation was analyzed in 2411 patients, and reoperation occurred in 86 patients (3.56%). When reoperation was compared between the groups, 60 of 980 patients (6.12%) from the repair group were reoperated, against 26 of 1431 patients (1.81%) from the replacement group^[10,11,14]^. Therefore, the reoperation rate was higher in the repair group.

### Stroke

Postoperative stroke was analyzed in 981 patients. The data revealed that there was no difference between the groups, with 10 cases in each one. One of the studies presented that all events were correlated with the mechanical prosthesis (*P*=0.048)^[10]^.

### Endocarditis

Results for infectious endocarditis (IE) were found in five studies, with 1230 patients analyzed. In one study, it varied from 3% in the preoperative period in the repair group to 1% in the same group in the postoperative period^[10]^. On the other hand, in the replacement group, between the pre and postoperative periods, it varied between 5% to 3%, most of them occurring with biological prosthesis^[10]^. In other studies, this data is not very clear.

### Mitral Stenosis

The appearance of postoperative mitral stenosis was increased in the repair group (3.1%) compared to the replacement group (0.9%). A retrospective cohort study compared mitral stenosis appearance between the groups with and without intervention, and there was no difference (14.4% × 16.5% [*P*=0.83])^[12]^.

### Mortality and Survival

The mortality rate was reported in three studies, accounting for 110 (3.83%) patients. Two articles^[10,14]^ demonstrated a statistically significant difference between the repair group and the replacement group, showing, respectively, values of 0.88% *vs.* 8.17% (*P*<0.001). Another study^[11]^ showed 0% mortality in the repair group and 4% (n=10) in the replacement group, but no *P*-value was presented.

One of the studies highlighted important factors that increase the mortality risk as NYHA III/IV, non-elective surgeries, and reoperations. Another study of larger samples indicated that the mortality in the MV replacement group is considerably higher than in the repair group (n=73 and n=11, respectively). Another study pointed out that the replacement by biological prosthesis has higher mortality compared to mechanical prosthesis and repair; these have similar rates with little discrepancy when compared.

Two cohorts showed that 292 of 368 patients were still alive after five years of follow-up in the repair group, on the other hand, 351 of 399 were alive in the replacement group. A cohort study showed a survival rate up to four years being 98 ± 2% with mechanical prostheses and 70 ± 10% with bioprosthetic ones (*P*=0.011)^[10]^.

## DISCUSSION

The literature shows that the recovery of the NYHA functional class from stages III-IV to I-II in the postoperative period was greater in the repair group than in the replacement group^[16]^, in addition to being indicated in patients with NYHA classes III-IV^[17]^. However, our study found that, currently, the percentage of recovery between the two groups was not significantly relevant, demonstrating close values.

A study compared the reoperation rate of biological and mechanical prostheses. However, only the biological valve presented this rate, being disregarded with the other prosthesis^[18]^. Crossing the data with another article, it is possible to infer that biological prosthesis presents the highest rate, considering younger patients and presenting a greater risk in elderly patients^[19]^.

Other variables such as thromboembolic events, especially stroke, in the first 30 days after surgery are lower with mechanical prosthesis, and considering the postoperative period after this gap, it equalizes with the biological prosthesis, unlike pulmonary embolic events which do not present such variation between the two approaches in the immediate and late postoperative periods^[18]^.

As a postoperative complication, IE is one of the variables observed in RHD follow-up^[20]^. In an Indian study, 277 patients, aged between 10 and 62 years, with RHD underwent double valve replacement - 12 patients developed IE, of which seven died, but all deaths had previous valve endocarditis^[18]^. Still on valve replacement, another study, with 1691 patients aged between 50 and 70 years, comparing biological and mechanical prostheses, had five and two cases, respectively, but the population in the biological replacement group was older and with more previous comorbidities^[21]^. However, in the study involving 54 patients, including children and adolescents, the repair was the procedure chosen for all, and only three patients had previous IE, with no new reports in the follow-up of the study^[19]^. Such data corroborate the greater number of cases with IE in valve replacement.

Bleeding is present in the analysis of two articles, with intracranial and gastrointestinal bleeding being the most common. A study shows that in the first six months, the rate of major bleeding is higher in the mechanical prosthesis group, but within one year compared to the biological prosthesis group these values begin to approach, showing in the study itself that it is not possible to state that one method presents a higher bleeding rate^[19]^. Although the other article demonstrated that even though the values are close, intracranial bleeding is still more present in mechanical exchange in proportion to other locations due to anticoagulation^[18]^. A third study showed that in repair, the bleeding rate is closely associated with other post-surgical conditions such as mitral stenosis and residual mitral insufficiency, however, there were few cases of major postoperative bleeding in this group even with the association of these conditions^[21]^.

According to the results of the present study, MV repair had more vantage than MV replacement concerning mortality rate, endocarditis, and stroke, but it had a higher rate of reoperation. Considering the diversity of the population in age and different settings of conditions and facilities worldwide, this study can aggregate all of these differences and give us an understanding of this doubt.

Our results reinforce that for the young population, repair is the best alternative because it presents lower mortality and greater survival, when the repair is adequate; and for the young adult population, it is noted that repair ends up being the best alternative, because even with a higher rate of reoperation compared to replacement, many patients will not need replacement; the mortality rate is still lower, and the survival rate is higher. Thus, we observed that for the general population, regardless of age, repair is the best surgical technique concerning replacement, both mechanical and biological.

A different meta-analysis shows results in agreement with the present study^[22]^. According to this article, MV repair reduces early mortality and improves long-term survival, and there were less valve-related adverse events, although it has been associated with an increased risk of reoperation^[22]^.

The considerations presented are supported by relevant guidelines. The Update of the Brazilian Guidelines on Valvular Heart Diseases^[17]^ states that patients with mitral stenosis of rheumatic etiology, without contraindications or complicating factors, should undergo mitral valvuloplasty via balloon catheter as first-line treatment; and, when unable to follow this technique, they must undergo labial commissurotomy and, only if it is impossible to maintain the native valve, change to the protheses approach - this sequencing of conduct corroborates the preference for repair over replacement.

Furthermore, the 2020 Guideline for the Management of Patients with Valvular Heart Disease from the American College of Cardiology^[23]^ updated the treatment approach for patients with primary mitral regurgitation. In this case, when the surgical treatment of mitral regurgitation of rheumatic origin is chosen, the repair is considered well-tolerated and durable. It is important to add that both guidelines present different approaches for patients with MV disease of degenerative origin.

Contrarily to these results, a retrospective cohort presented that repair does not have considerable differences in mortality, survival, follow-up, and complications. However, the reoperation rate differs, and it had to be considered since the repair has a higher incidence, so variables such as age, life expectancy, and previous morbidities are essential considering that the patient might not be able to reoperate in the future^[24]^.

When the replacement technique is adopted, an article uses age as the main parameter to choose between mechanics and bioprosthetics, demonstrating that despite the tendency of bioprostheses for younger patients, mechanical replacement of the MV may be a more reasonable alternative in patients over 50 years old^[18]^.

Regarding the cost of each surgery, repair is cheaper, with an average value of US$ 2530, while biological valve replacement (US$ 3190) and mechanical valve replacement (US$ 3220) are more expensive, following unadjusted values from 2018; however, QALYs were higher in biological (10.1), followed by repair (9.7), and finally in mechanical (6.2) replacement^[6]^. Therefore, for individualized and multidisciplinary conduct, it is pertinent to evaluate both cost and the quality of life, considering the financial condition of each health system, and valuing the best outcome for the patient.

To analyze the risks of bias in these studies, two main characteristics were observed in each one: how the statistical analyses were performed and what the limitations of the studies were. In addition, we use the Cochrane Collaboration tool, which covers sequence generation, allocation concealment, blinding, incomplete result data (*e.g.*, dropouts and withdrawals), and selective results reporting.

### Limitations

As limitations, we can highlight a small number of selected studies, both due to the strict inclusion and exclusion criteria and the lack of case controls and cohort studies that would contemplate our analyses. Furthermore, the heterogeneity of the data studied and the majority of our study population being located in only one country limit our study, and there was no meta-analysis of the data.

## CONCLUSION

In conclusion, MV repair demonstrated lower mortality, complications, and a similar reoperation rate compared with MV replacement. The survival was similar between the groups.
